# Supplementary material for: Dimensional hierarchy of higher-order topology in three-dimensional sonic crystals
Source: Nat Commun. 2019 Nov 25;10:5331. doi: 10.1038/s41467-019-13333-9 (PMC6877633; doi:10.1038/s41467-019-13333-9)
Supplement: Supplementary file 1 — Supplementary Information [file 41467_2019_13333_MOESM1_ESM.pdf]

Supplementary Information

Dimensional hierarchy of higher-order topology in three-dimensional sonic crystals

Xiujuan Zhang[1*], Bi-Ye Xie[1*], Hong-Fei Wang[1*], Xiangyuan Xu[1, 3], Yuan Tian[1], Jian-Hua Jiang[2†], Ming-Hui Lu[1, 4, 5†] & Yan-Feng Chen[1, 5]

[1]*National Laboratory of Solid State Microstructures, Department of Materials Science and Engineering, Nanjing University, Nanjing 210093, China*

[2]*School of Physical Science and Technology, and Collaborative Innovation Center of Suzhou Nano Science and Technology, Soochow University, 1 Shizi Street, Suzhou 215006, China*

[3]*Key Laboratory of Noise and Vibration Research, Institute of Acoustics, Chinese Academy of Sciences, Beijing 100190, China*

[4]*Jiangsu Key Laboratory of Artificial Functional Materials, Nanjing 210093, China*

[5]*Collaborative Innovation Center of Advanced Microstructures, Nanjing University, Nanjing 210093, China*

[†]Correspondence and requests for materials should be addressed to jianhuajiang@suda.edu.cn (JHJ) or luminghui@nju.edu.cn (MHL)

* *These authors contributed equally to this work.*

Supplementary Note 1: Topological phase transition in the sonic crystals

The phase transition between the topological phase and the trivial phase is signaled by a gap-closing-reopening process, accompanied by a band inversion. In Fig. 1 of the main text, we have presented the band structures for the topological and trivial sonic crystals (SCs). They share the same band gap but distinct topological properties, characterized by the band inversion. To further see this, here we provide a study on the band evolution by deforming the lattice. In the main text,

we emphasized that the geometric parameters of the SCs are carefully tuned such that our system is experimentally feasible and measurable while maintaining a large band gap. In doing so, we have set the cavity width as $w_c = 6$ mm, the channel width as $w_t = 3$ mm and the deforming distance (i.e., the shrinking or expanding distance) as $d_{off} = \pm 3.5$ mm ($d = 0.25a + d_{off}$ with $a = 2$ cm). The geometric parameters w_c, w_t and d are defined in Supplementary Figure 1, whose values are consistent with those illustrated in Fig. 1 of the main text. In the non-deformed case, i.e. $d_{off} = 0$, the eight cavities will overlap with each other. In order to better characterize the topological transition in our system, we choose another design with $w_c = 4$ mm (the other geometric parameters are the same as that used in the main text), whose lattice configurations are sketched in Supplementary Figure 1a-c, respectively for the shrunken, non-deformed and expanded lattices. Their corresponding bulk band structures are also presented, clearly showing a band gap-closing and reopening process when deforming the lattice. The band gap closing happens exactly when the lattice is not deformed (see Supplementary Figure 1b), while the band gap opening happens when the lattice is either shrunk (Supplementary Figure 1a) or expanded (Supplementary Figure 1c), similar as that studied for the lattice design with $w_c = 6$ mm (Fig. 1 in the main text). Essentially, these two designs carry the same physical properties. More evidence can be found in Supplementary Figure 1d where the similar field maps as that in the insets of Fig. 1 are presented, only for the eigen-states at the lowest bands in Supplementary Figures 1a and 1c. Again, we observe similar parity properties as that in Fig. 1. At the Γ point, the parities for the shrunken (trivial) and expanded (topological) lattices are the same, while they exhibit opposite parity orders at the X, M and R points.

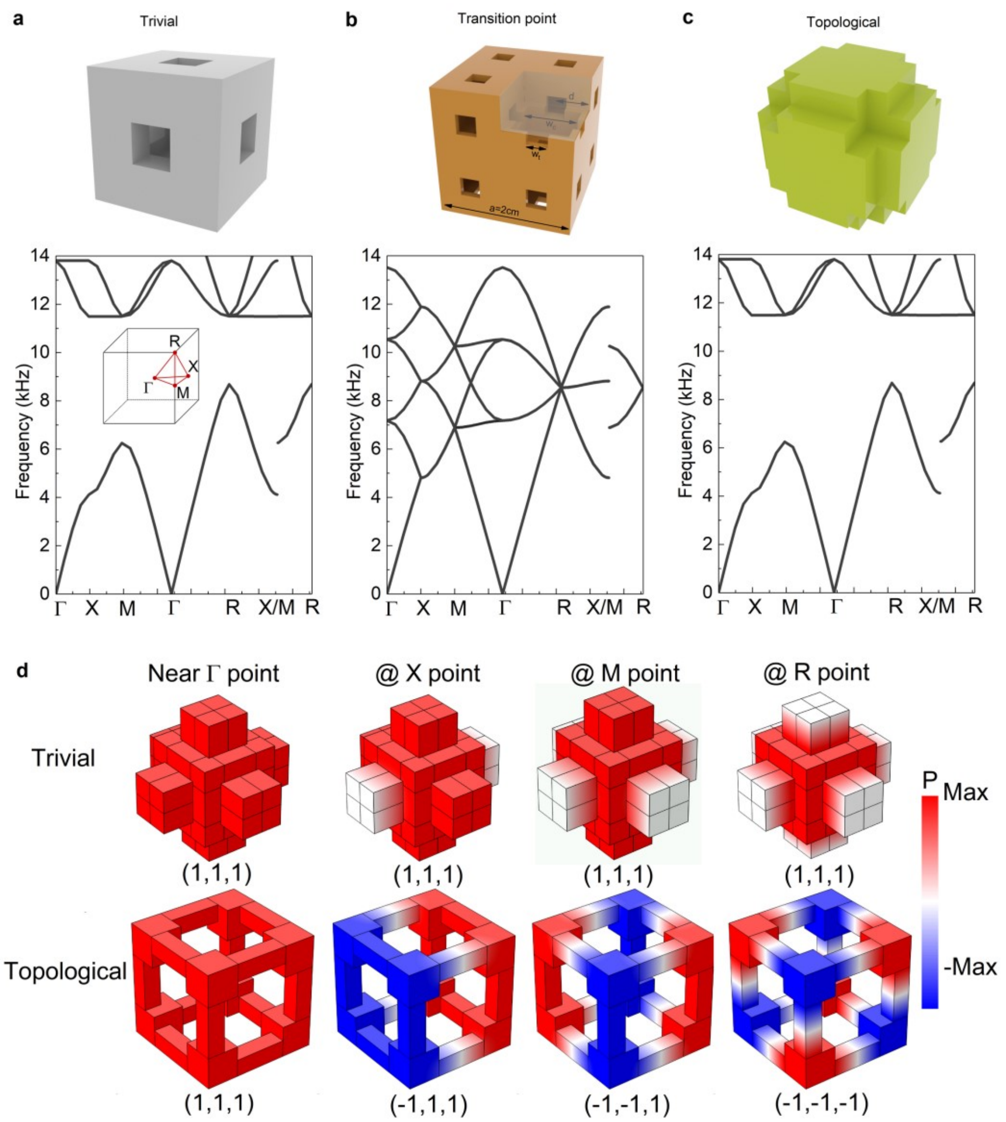

Supplementary Figure 1 | Band evolution and parity properties for the lattice design with $w_c = 4$ mm. a-c, Respectively the sketched unit cells for the shrunken (trivial), non-deformed and expanded (topological) lattices, as well as their corresponding bulk band structures. **d,** Pressure field patterns for the eigen bulk states located at the Γ, X, M and R points for the lowest bands in **a** and **c**. The upper panel indicates the trivial phase and the lower panel indicates the topological phase. The eigen-values of the three mirror symmetries for each eigen-state are also labeled.

Similar behaviors as that in Fig. 1 are observed, where the states at the Γ point have the same parity for the trivial and topological phases, while the states at all the other high symmetric points (the X, M and R points) have opposite parity orders.

Supplementary Note 2: Bulk topology in 3D systems based on the Wannier center description

Based on the modern polarization theory [1,2], the bulk topology of our SCs can be described by the positions of the Wannier centers (WCs) which are pinned to the maximal Wyckoff positions (which are illustrated in Supplementary Figure 2a) [3,4] due to the mirror symmetries in our system. In fact, the vector sum of the center of the WCs is related to the macroscopic polarization of a crystalline insulator [5]. For a unit cell, the local displacement of a WC from the origin point represents a microscopic map of the local polarization field. Then the sum of these local polarization fields leads to the macroscopic quantum mechanical polarization of the system.

For topologically trivial insulators that are topologically equivalent to the atomic insulators, the WCs are pinned to the center of the unit cell as shown in Supplementary Figure 2b and there is no local polarization field. On the contrary, topologically non-trivial insulators have WCs that are away from the center of the unit cell (see Supplementary Figure 2c-e), which are denoted as the obstructed atomic insulators. When we consider the three mirror symmetries, there are three cases where the WCs can stay at the centers of the surfaces (Supplementary Figure 2c), the middle points of the hinges (Supplementary Figure 2d) and the corners (Supplementary Figure 2e). The augments of these local polarization fields result in the first-, second- and third-order topology in the topological crystalline insulators.

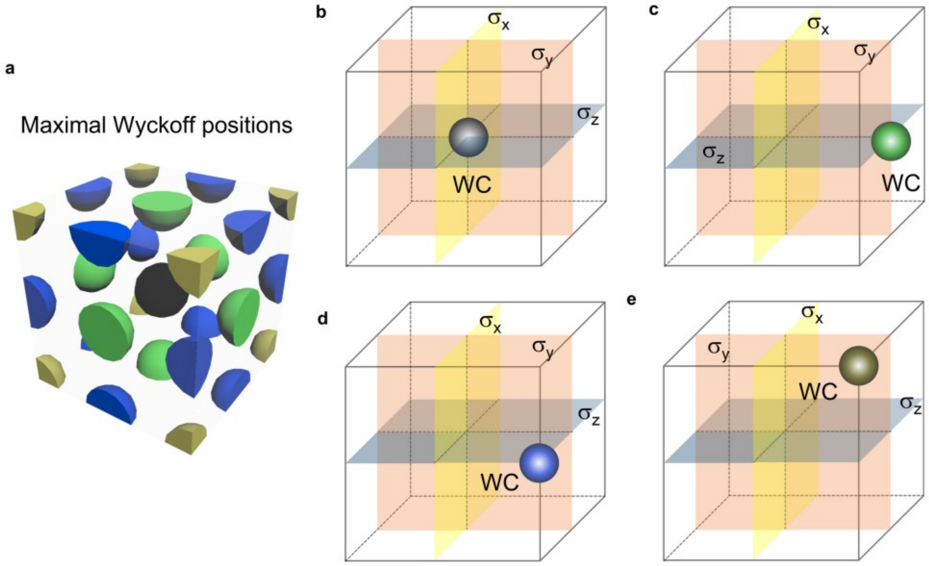

Supplementary Figure 2 | Wannier center description on the bulk topology in 3D systems. The Wannier centers (WCs) are indicated by the colored spheres. a, The maximal Wyckoff positions in the designed simple cubic SCs. **b,** WC at the center of the unit cell, where there is no local displacement and polarization field. This represents a trivial topological insulator. **c-e,** WC at the center of the surface, middle point of the hinges, and the corners of the unit cell, respectively, representing a topological insulator with first-, second- and third-order topology.

Supplementary Note 3: Pressure field distributions for the eigen surface states

In Fig. 2a of the main text, we present the projected band structure of a ribbon-like supercell and find the surface states emerging in the gap of the bulk states. To further confirm those in-gap states are indeed surface states, in Supplementary Figure 3 the pressure field distributions of the eigen-states marked in the band structure are plotted, which exhibit typical behaviors of the surface states,

i.e., the field is mainly confined on the interface between the topological and trivial lattices. When going away from the interface, the field intensity decays rapidly.

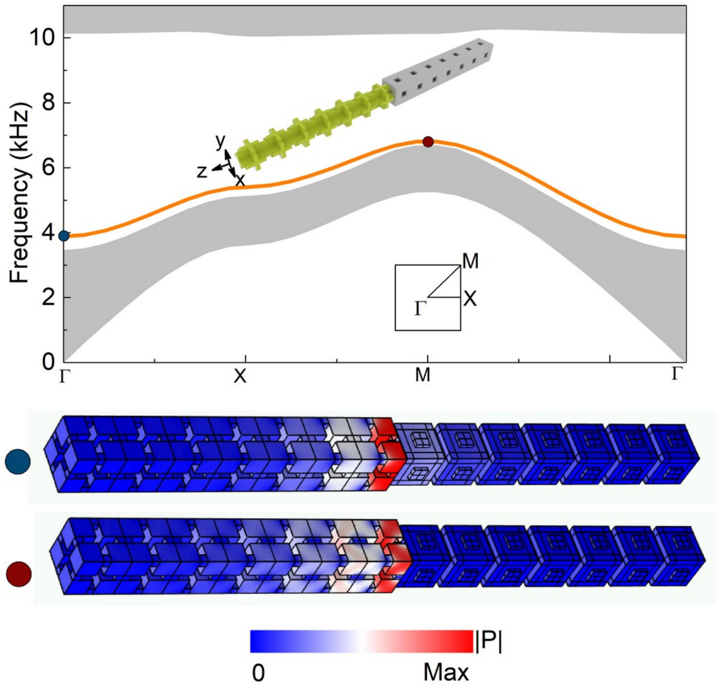

Supplementary Figure 3 | Pressure field distributions of the eigen surface states. The same projected band structure as that in Fig. 2a of the main text is presented, where the in-gap states are found (the orange curve). Two of them (one at the Γ point and the other at the M point) are particularly highlighted, whose pressure field distributions are presented. It is observed that the pressure field is mainly concentrated on the interface, consistent with the properties of the surface states.

Supplementary Note 4: Simulations on the excitation of the surface states

In our system, the surface states can be independently excited, despite that they share almost the same frequency range with the bulk states. To provide more evidence, we present in

Supplementary Figure 4 the simulations on the excitation of the surface states. We use the same set-up as the experiments (see Fig. 2c in the main text), only in the simulations a source with varied location is used. We find that the surface states can always be excited with a much higher power level than the bulk state, even for the cases where the source is put inside the bulk, indicating the surface states have higher density of states and can be independently excited.

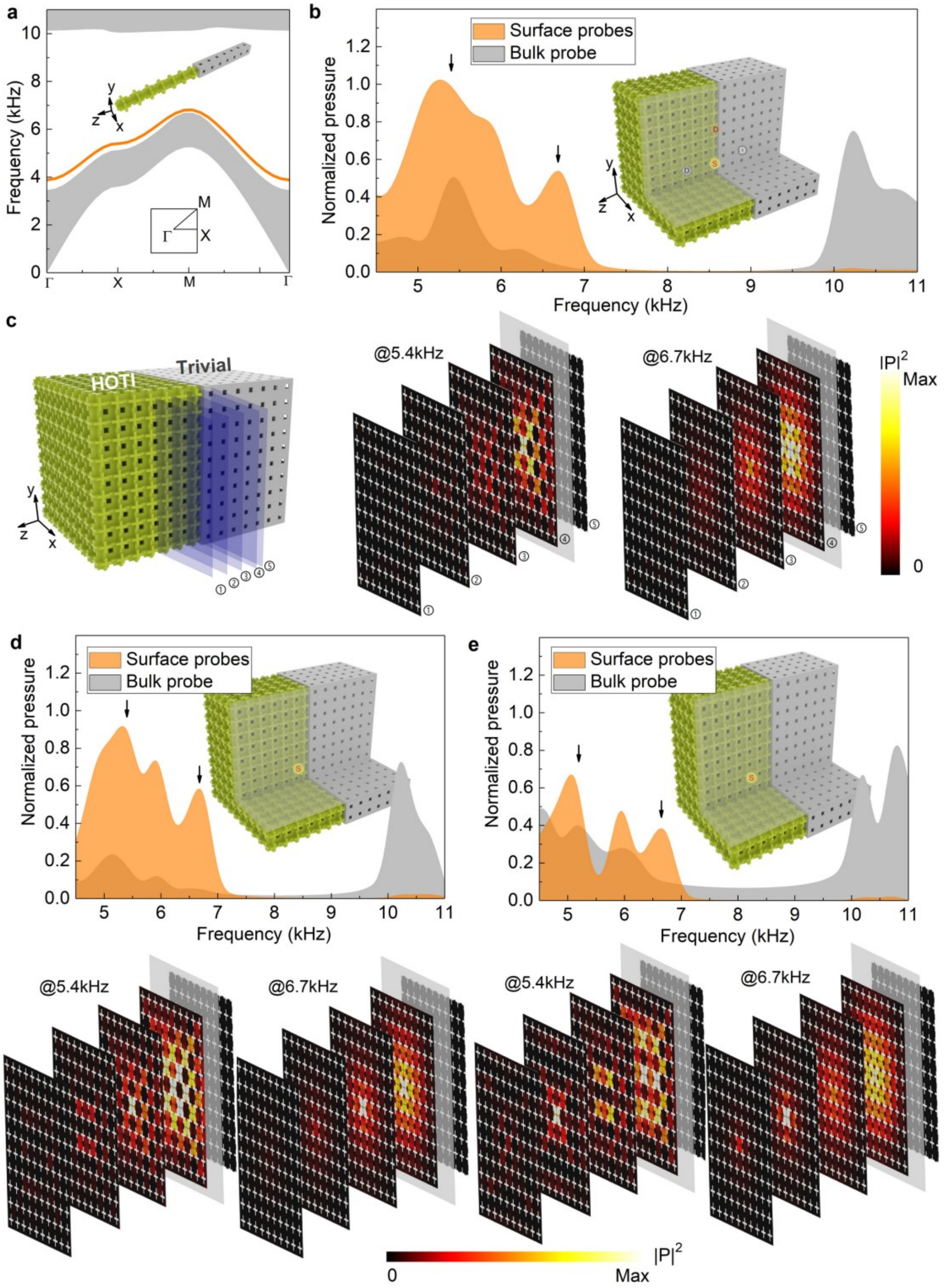

Supplementary Figure 4 | Excitation of the surface states in simulations. a, Same projected band structure as that in Fig. 2a of the main text. **b,** Same transmission spectra as that in Fig. 2c of the main text, only the data are obtained from the

simulations. The same set-up as the experiment is used, where the source and probe locations are highlighted in the inset. **b,** Simulated field maps for the excited surface states at 5.4 kHz and 6.7 kHz, where the surface probe finds excitation peaks (as denoted by the black arrow in **b**). Notice that at both operating frequencies, the excited modes exhibit typical behaviors of the surface states, i.e., the energy is mostly concentrated on the topological/trivial interface and decays rapidly when going into the bulk region. **d and e,** Same as **b**, but with source located in the bulk region (the topological lattice side). The source locations are marked in the insets. It is seen that the surface probes still receive most of the energy even when the source is placed in the bulk, indicating that the surface states have higher density of states than the bulk states and can always be excited independently. The same field maps as **c** are also provided, which show the similar behaviors and suggest that the excited modes are indeed surface states.

Supplementary Note 5: Study on the degeneracy of the hinge and surface states

Figure 3a of the main text presents the projected band structure of a slice of the hinge sample, which gives two types of in-gap states: the hinge and surface states. We show in Supplementary Figure 5 the frequencies of the eigen-states at $k_z = 0$ as functions of the solution number and find that there are eight states emerging in the gap of the bulk states. Further simulations on the pressure field distributions confirm that four of them are hinge states and the other four are surface states. Each of these four states are degenerate in terms of their emerging frequencies.

Moreover, we would like to point out that there are only four surface state emerging in this hinge supercell, which otherwise are expected to be dispersive along both k_x and k_y directions of the surfaces. This is because the topological sonic crystal in our hinge sample consists of 8×8 unit cells (surrounded by a layer of trivial sonic crystal with 4 unit cells). Limited by the computational power, such a small size scale can only allow the existence of the fundamental surface states (i.e., the surface states with the smallest values of possible k_x and k_y) and hence there are only four degenerate surface states observed in our system, consistent with the four interfaces constructed between the topological and trivial sonic crystals. If we increase the sample size (e.g., the number of unit cells in the topological sonic crystal becomes 12×12), it is found that the surface states with higher-k begin to emerge and they may also interact with the lower-k components of the surface states. As a result, the surface states occupy a finite width in frequency (see Supplementary Figure 6). Meanwhile, the hinge states remain to be four degenerate states localized on the hinges, as shown by the pressure field maps presented in Supplementary Figure 6.

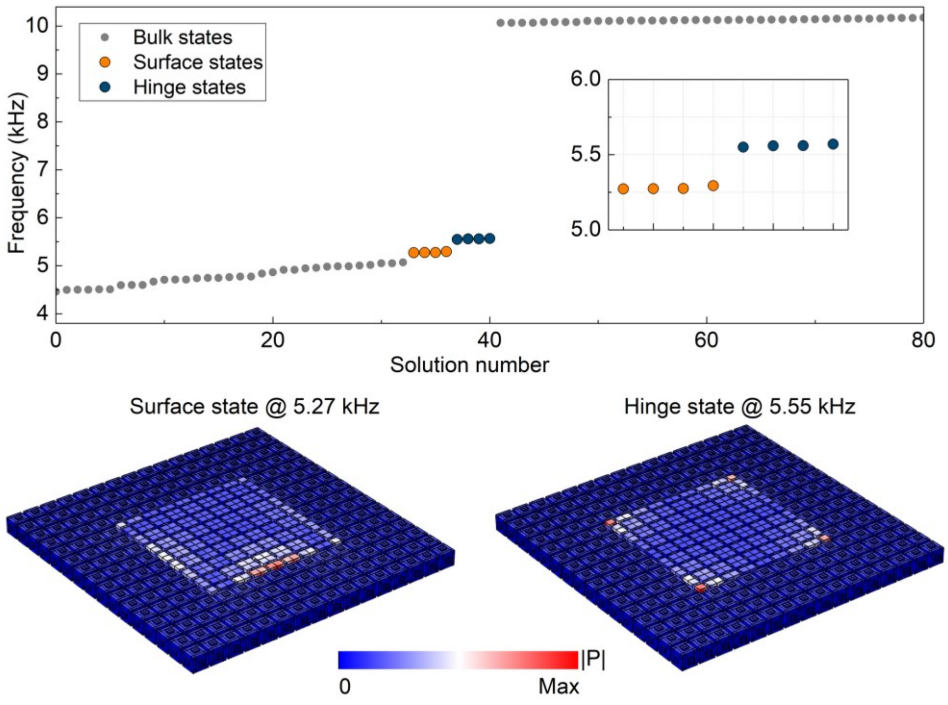

Supplementary Figure 5 | Degeneracy study on the hinge and surface states. Frequencies of the eigen-states at $k_z = 0$, calculated for the hinge supercell (which is depicted as the inset in Fig. 3 of the main text), as functions of the solution number are plotted. Eight in-gap states (colored) are observed, each four of them are degenerate (an enlarged figure further confirms this). The pressure field distributions of two of these in-gap states (one belongs to the orange group and the other belongs to the dark blue group) are presented, which exhibit the typical behaviors of a surface state and a hinge state.

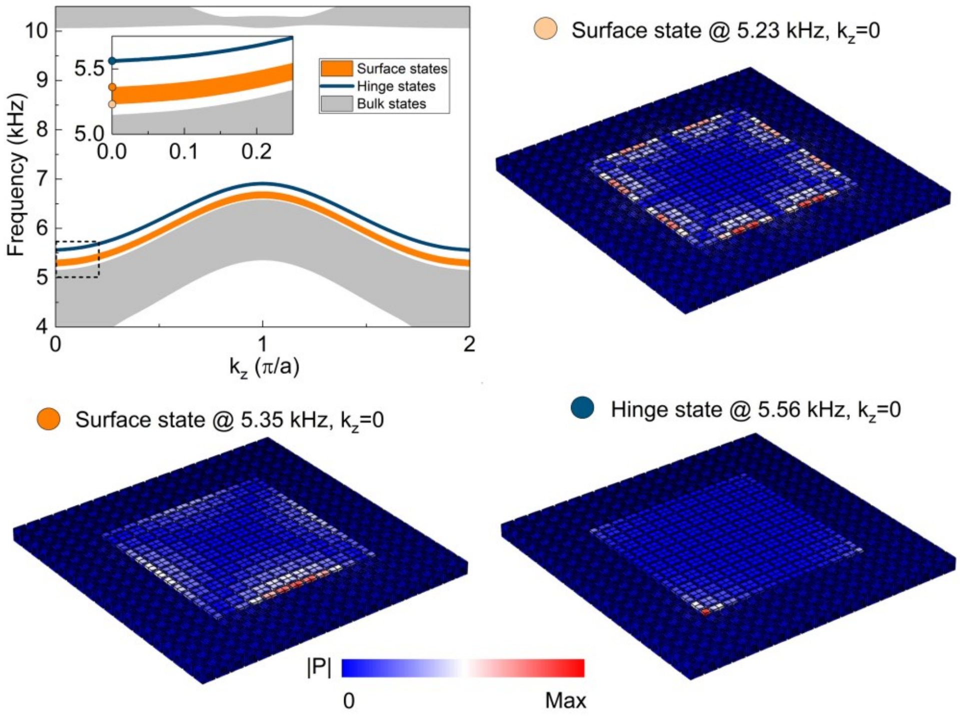

Supplementary Figure 6 | Topological hinge and surface states in the hinge supercell with 12 × 12 unit cells. The calculated eigen spectra of the hinge supercell are presented. The simulation set-up is the same as that in Fig. 3a of the main text. It is seen that in this sample, the surface states occupy a finite frequency

range, different from the case in the smaller sample (i.e., the 8×8 unit-cell sample presented in Fig. 3a of the main text) where only four degenerate surface states are found. This is because the present sample supports both fundamental and higher-k surface states, which are split in frequency, as shown by the surface state field maps (represented by the light and dark orange dots). One surface state locates at 5.23 kHz, possessing the higher-k components, while the fundamental surface state locates at 5.35 kHz whose field exhibits a Gaussian profile along the direction parallel to the surface. On the other hand, the hinge states remain to be four degenerate states localized on the hinges, as shown by the field map indicated by the dark blue dot (here, only one hinge state is presented).

Supplementary Note 6: Surface state measurements in the hinge sample

In the main text, Fig. 3 presents our measurements on the hinge states in a sample composed of a block of expanded (topological) lattice surrounded by the shrunk (trivial) lattice. We refer to this sample as the hinge sample. From Fig. 3, it is observed that the system supports topological boundary states located on the hinges of the sample, just as we theoretically predicted. In addition to the hinge states, we also expect surface states in this sample, as shown by the simulations on the projected band structures of a slice of the hinge sample in Fig. 3a of the main text or Supplementary Figure 7a. To further visualize the surface states in the hinge sample (a photo of this sample is depicted in Supplementary Figure 7b), we conduct another measurement by putting the excitation source near to the surface (the source position is marked in the inset of Supplementary Figure 7c). The corresponding transmission spectra probed at different locations (which are also marked in

the inset of Supplementary Figure 7c) are presented in Supplementary Figure 7c using the dark blue color indicting the hinge probe, the orange color indicating the surface probes and the gray color indicating the bulk probe. Again, we observe the strong excitation power level for the hinge probe around 5.7 kHz, consistent with that in Fig. 3c in the main text. Additionally, we also observe a strong excitation peak around 4.7 kHz for both the hinge and surface probe, while the bulk probe is relatively low, indicating the excitation of the surface state. We further present the measured field distribution for this surface state, as shown by the cut-off slices plotted in Supplementary Figure 7d. The corresponding locations of the cut-off slices are illustrated in Supplementary Figure 7b. It is clearly seen that the excited mode is mostly concentrated on the two interfaces between the topological and trivial lattices along *x-z* and *y-z* planes. While going into the bulk, the waves are rapidly decayed.

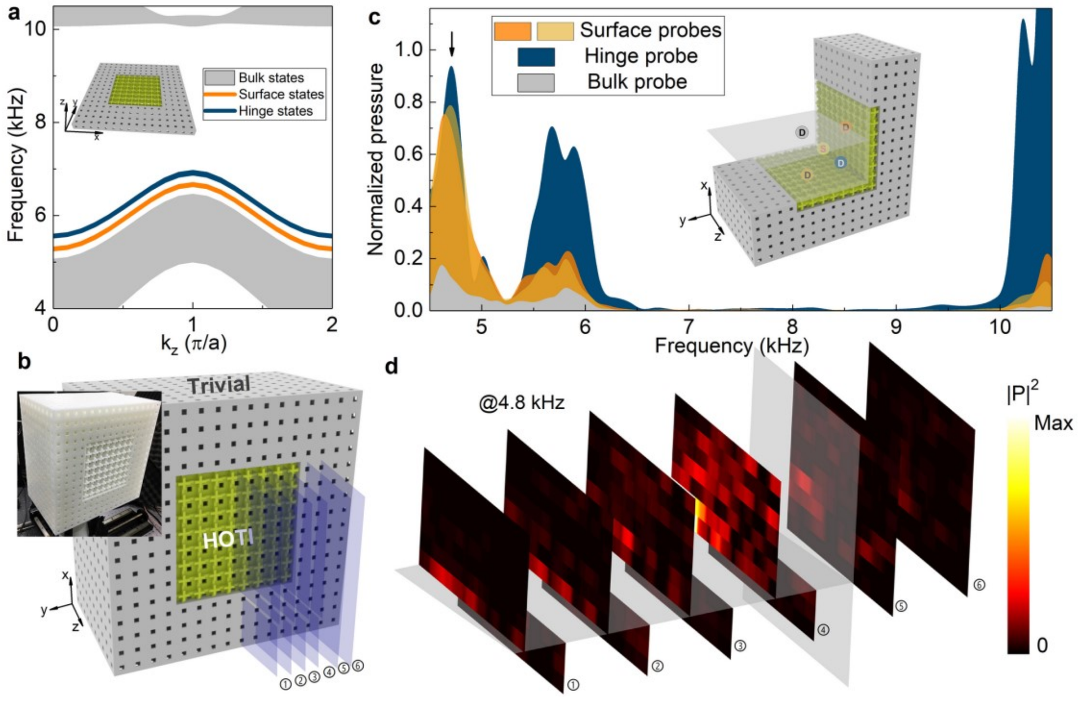

Supplementary Figure 7 | The surface state measurements in the hinge sample.

a, Projected band structure simulations for a slice of the hinge sample (which is

illustrated by the inset). Notice that both the hinge and surface states are found. **b,** A photo of the hinge sample. The sketch of the hinge sample is also provided, along with the cut-off slices indicating the locations where the experimental measurements are conducted. **c,** Transmission spectra measurements when an acoustic point source is injected. The source and probe locations are highlighted in the inset by the colored markers. We conduct the hinge (indicated by the dark blue color), surface (orange color) and bulk (gray color) probes. Notice that in addition to the excitation of the hinge state around 5.7 kHz, a surface state excitation is also observed around 4.8 kHz (denoted by the black arrow in **c**), whose field distributions are measured and plotted using the cut-off slices in **d**. Typical behaviors of a surface state, i.e. the energy is mostly concentrated on the interfaces between the topological and trivial lattices, are clearly observed, indicating the excitation of the surface state.

Supplementary Note 7: The complete corner sample and its cut-off

A corner sample consisting of a block of topological SC with $8 \times 8 \times 8$ cells enclosed by the walls of the trivial SC with the thickness of 4 cells is fabricated, whose photography is shown in Supplementary Figure 8. In order to be experimentally measurable, which requires the detector get into the sample and probe the pressure field on demand, we deliberately cut off two slices of the trivial lattice such that the topological lattice is exposed. The cut-off is performed along the indicating lines shown in Supplementary Figure 8. The resulted corner sample is shown in Fig. 4a of the main text.

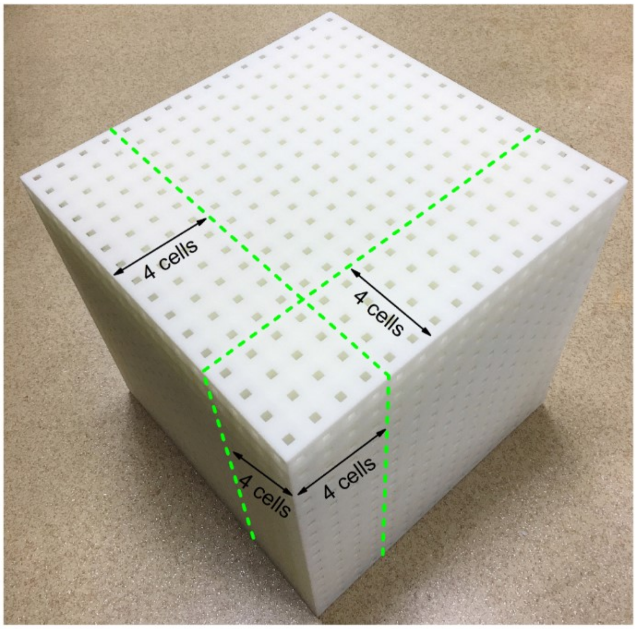

Supplementary Figure 8 | The complete corner sample and its cut-off procedure. A photograph of the complete corner sample is presented, with the green lines and the size notes indicating how the cut-off is performed.

Supplementary Note 8: Hinge and surface states measurements in the corner sample

As predicted by the theory, in the corner sample, we have not only the corner states, but also the hinge and surface states. This is the manifestation of the third-order topology, similar as that in the hinge sample, where the second-order topology manifests itself as the concurrent emergence of the surface and hinge states. To further demonstrate this, here we present the measurements of the hinge and surface states in the corner sample. As presented in Fig. 4 in the main text, we have fabricated a corner sample where a block of expanded (topological) lattice is enclosed by the shrunk (trivial) lattice to form eight corners, only that two slides of the trivial SC are removed for the sake of experimental measurements. From the transmission spectra in Fig. 4b in the main text, we observe the corner state excitation around 7.9 kHz, along with the hinge state excitation around

5.7 kHz, the surface state excitation around 4.7 kHz and the bulk excitation around 3.6 kHz. In the main text, we have presented the field distributions of the corner state, which exhibits a typical behavior of concentrating at the corner while quickly decaying on the hinge, surface and in the bulk. Here, in addition to the corner state measurement, we also present the field maps measured for the hinge and surface states, respectively in Supplementary Figures 9a and 9b. Similar as that in Fig. 4c in the main text, a series of cut-off slices are plotted to see the field distributions of the excited modes. In Supplementary Figure 9a, it is observed the excited mode is mostly concentrated on the hinges, unlike that in Supplementary Figure 9b where the excited mode is mostly concentrated on the surfaces, especially on the *y*-*z* plane (the source is placed closer to this plane).

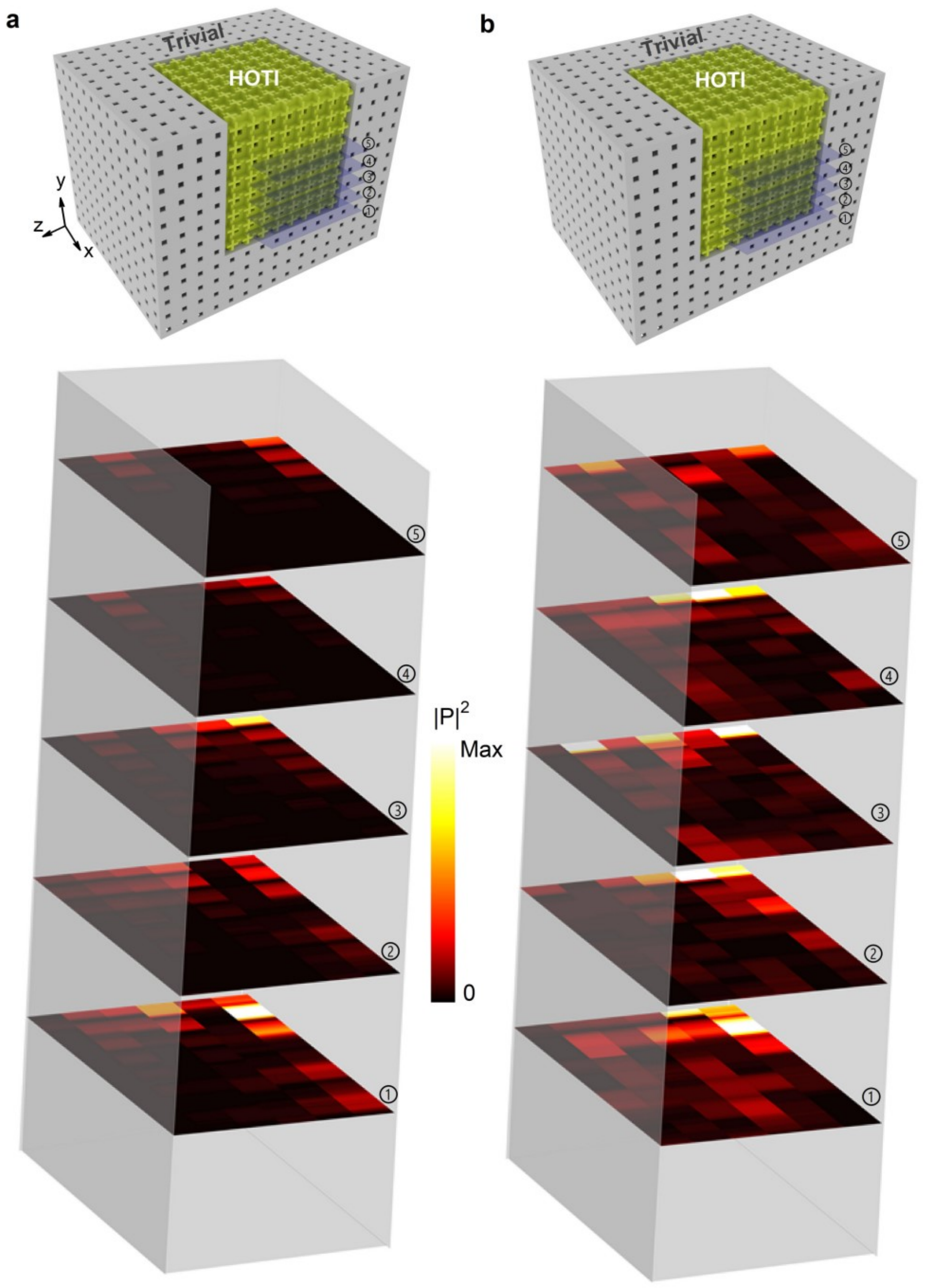

Supplementary Figure 9 | The hinge and surface states measurements in the corner sample. a and b, Respectively the measured field distributions for the hinge and surface states excited around 5.7 kHz and 4.7 kHz. The cut-off slices are shown to indicate the locations where the experimental measurements are conducted. The set-ups for the measurements are the same as that used in Fig. 4 in the main text.

Supplementary Note 9: Comparison between the topological corner states and the trivial cavity modes

In this section, we compare the topological corner states in our system with the trivial cavity modes to demonstrate the robustness of the former. For this purpose, we deliberately create a cavity in the trivial lattice by replacing one cell with an air cavity whose geometric sizes are tuned such that there exists a cavity mode in the band gap of the trivial SC. The insets in Supplementary Figure 10a-b illustrate the cross-sections of the two structures that support the corner state (Supplementary Figure 10a) and the cavity mode (Supplementary Figure 10b). As theoretically predicted and experimentally observed in the main text, the corner state emerges at the corner of the non-trivial lattice truncated by the trivial lattice and is a manifestation of the non-trivial bulk topology, hence it has topological nature and protected by the bulk topology. On the other hand, the cavity mode appears as a defect mode whose origin is the resonance of acoustic waves inside that cavity and has no topological nature. Therefore, the cavity mode is not topologically protected.

To provide further evidence, we numerically study the robustness of the corner state and the cavity mode by considering the effects of shrinking the corner-cell/cavity-cell (Supplementary Figure 10c-d), removing the corner-cell/cavity-cell (Supplementary Figure 10e-f) and adding extra defects around the corner/cavity (Supplementary Figure 10g-h). Specifically, we inject an acoustic point-like source near the corner/cavity and probe the transmission signals at the vicinity of the corner/cavity and in the bulk of the trivial lattice. The obtained results are presented as functions of frequencies that are normalized with respect to the frequencies of the corner state and the cavity mode without any distortion (see Supplementary Figure 10a-b). The field maps for the corner state and the cavity mode are also presented corresponding to each probe. It is seen that the topological corner state has a rather small frequency shift (<3%) upon various distortions while the trivial

cavity mode is quite sensitive to the distortions (with frequency shift ~10%). Especially, when the cavity cell is removed, the cavity mode shifts by 14% on frequency (Supplementary Figure 10f). In addition, removing the neighboring cell from the trivial cavity configuration creates an extra defect and thus induces another defect mode (indicated by the blue arrow in Supplementary Figure 10h), while the extra defect only affects the corner mode by shifting its frequency by 2% in the topological corner configuration. These results again indicate that the topological corner state is indeed more robust than the trivial cavity mode, which may inspire new route to design robust lower-dimensional waveguides.

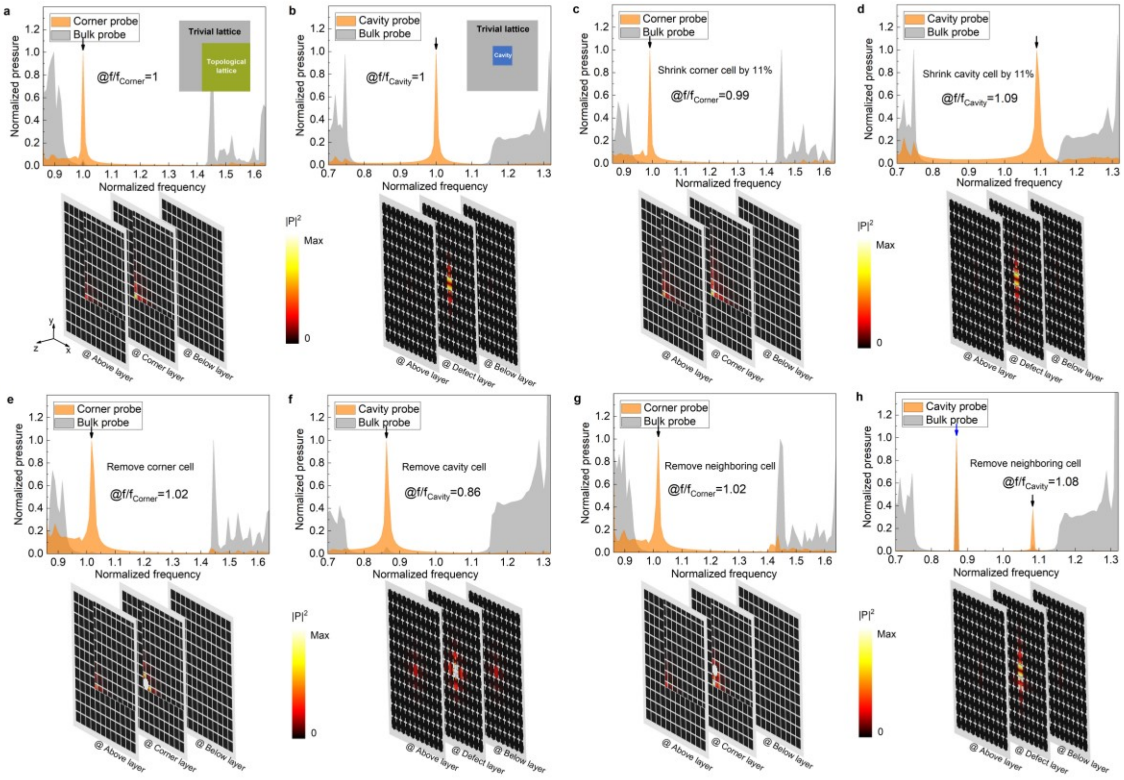

Supplementary Figure 10 | Comparison between the topological corner state and the trivial cavity mode. a and b, Respectively the simulated transmission spectra for the corner state and the cavity mode (whose geometric configurations are illustrated in the insets). The field maps for the excited corner state and the cavity

mode (whose frequencies are indicated by the black arrows) are also presented. Various defects are introduced to study the robustness of the corner state and the cavity mode, including shrinking **b,** the corner and **c,** the cavity cell by 11%, removing **d,** the corner cell and **e,** the cavity cell, and, removing **g,** the corner-neighboring cell and **h,** the cavity-neighboring cell. The similar transmission spectra as that in **a** and **b** are presented, with frequencies normalized by the frequencies of the corner state and the cavity mode without any distortions. The blue arrow in **h** indicates the defect mode induced by removing the cavity-neighboring cell (which creates another defect).

Supplementary References

[1] R. D. King-Smith and D. Vanderbilt, *Phys. Rev. B* **47**, 1651–1654 (1993)

[2] R. Resta, *Rev. Mod. Phys* **66**, 899–915 (1994)

[3] B. Bradlyn, L. Elcoro, J. Cano, M. G. Vergniory, Z. Wang, C. Felser, M.I. Aroyo and B.A. Bernevig, *Nature* **547**, 7663 (2017)

[4] W.A. Benalcazar, T. Li and T.L. Hughes, *arXiv preprint arXiv:1809.02142 (2018)*

[5] N. Marzari, I. Souza and D. Vanderbilt, D. *Psi-K newsletter* **57**, 129 (2003)
